# Supplementary material for: Neuronal messenger ribonucleoprotein transport follows an aging Lévy walk
Source: Nat Commun. 2018 Jan 24;9:344. doi: 10.1038/s41467-017-02700-z (PMC5783941; doi:10.1038/s41467-017-02700-z)
Supplement: Supplementary file 1 — Supplementary Information [file 41467_2017_2700_MOESM1_ESM.pdf]

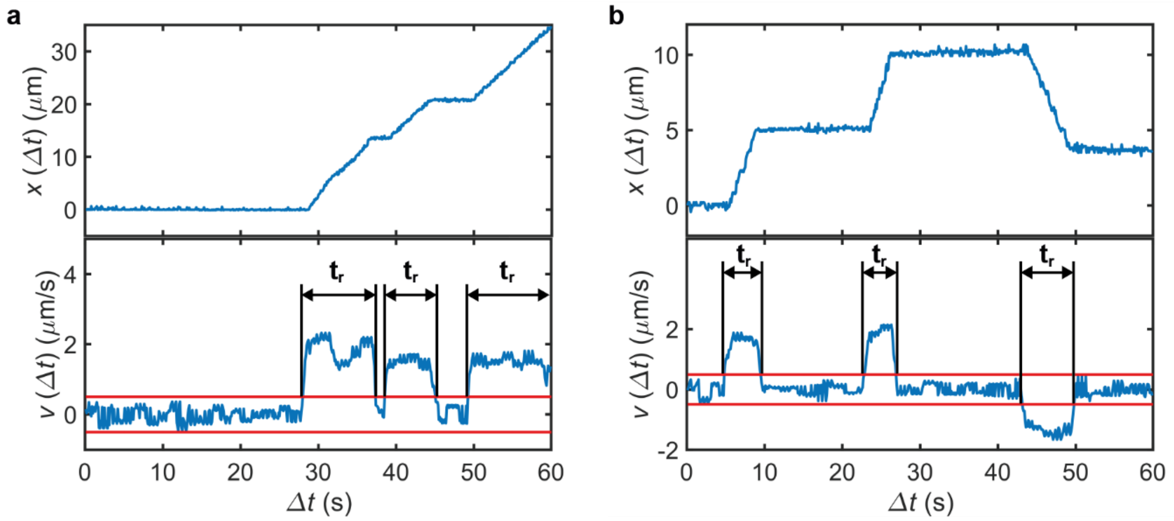

Supplementary Figure 1. Two examples of mRNP trajectories obtained in the experiment (upper panels) and the classification of run and rest phases from the corresponding trajectories (lower panels). In the lower panels, the velocity at each time point of trajectory is plotted and red lines represent the threshold value for distinguishing the motion of mRNP (i.e., run or rest). Details of methods for distinguishing the motion is in “Image analysis” in the methods section. The regions in the run phase are expressed with t_r in the lower panels and the other regions represent rest. Both run and rest phases are noisy because of the positional fluctuations with similar amplitudes. The noise is likely to be caused by the thermal motion or localization uncertainty for particle detection.

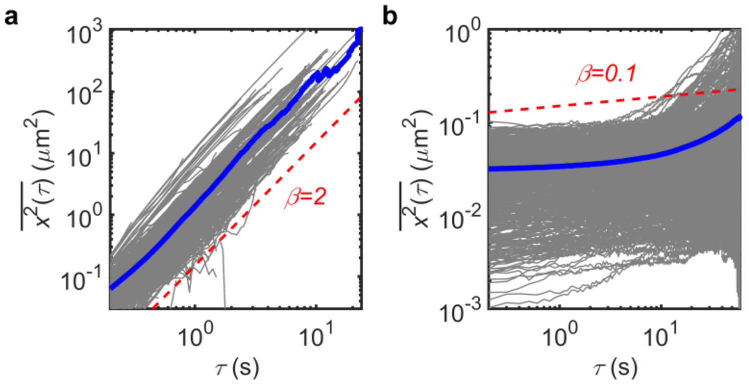

Supplementary Figure 2. Analysis of the mean squared displacement (MSD) for run and rest phases. From the procedure explained in Supplementary Fig. 1, the kymograph trajectories were segmented into two parts of run and rest, respectively. For each segment (of either run or rest phase), its MSD was evaluated by the method of self-averaging called time-averaged (TA) MSD by the definition $\overline{x^2(\tau)} = \frac{1}{T-\tau}\int_0^{T-\tau}[x(t+\tau) - x(t)]^2 dt$. **a.** TA MSD curves from 312 individual runs (gray) and its average curve (blue). The results show that run is a phase of ballistic motion with the MSD exponent $\beta \approx 2$. **b.** TA MSD curves from 562 rest segments (gray) and its average curve (blue). Rest phase exhibits a subdiffusive dynamics with the MSD exponent $\beta \approx 0.1$.

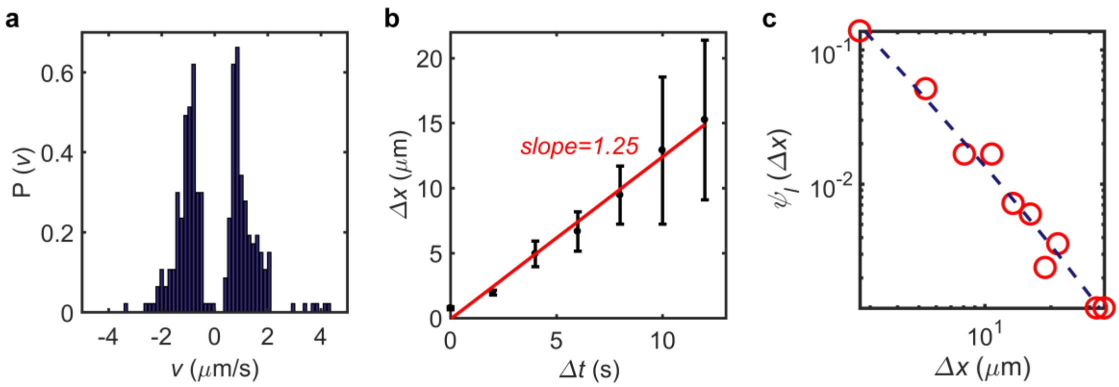

Supplementary Figure 3. Analysis of the dynamic properties of run phase. **a.** The distribution of run velocities. The velocity was evaluated as $v = \Delta x/\Delta t$ where $\Delta x, \Delta t$ are respectively the displacement and the duration of each run. The distribution is almost symmetric with respect to the direction and has peaks around at 1 μm/s. **b.** The average run length (Δx) as a function of the run time (Δt). The linear relation is found, indicating that run can be regarded as a ballistic motion of a constant speed (~ 1.25 μm/s). **c.** The distribution of run lengths. The dashed line represents the best fit to the data with $\psi_l(x) \propto \frac{\exp(-x/x_r)}{(x+x_0)^{1+\eta}}$. Its exponent η is ~0.81 and the characteristic length is ~86 μm.

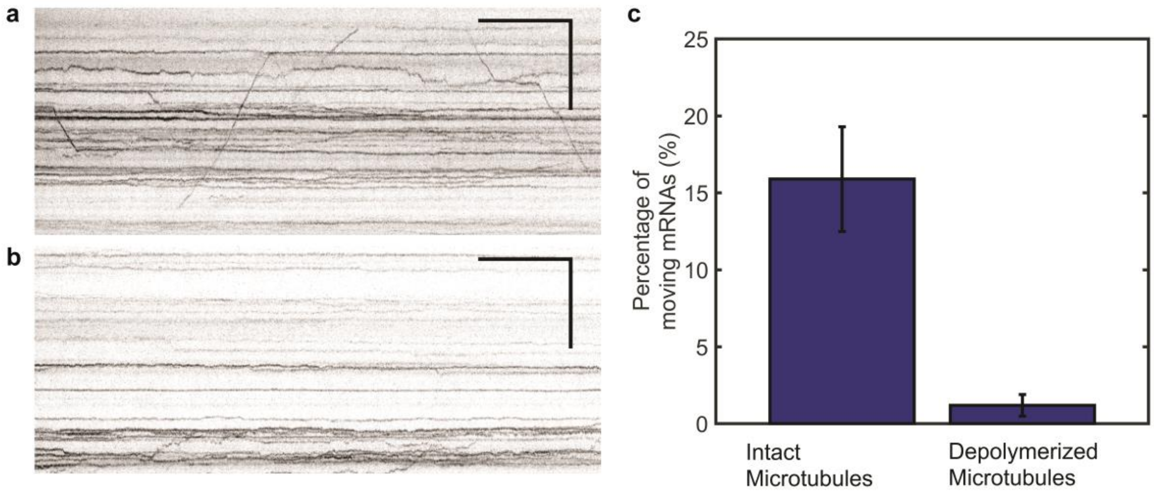

Supplementary Figure 4. Intact microtubules are needed for directed motion of mRNPs. **a**. A representative kymograph of mRNPs before nocodazole treatment. Some of mRNPs are showing directed motion. **b**. A representative kymograph of mRNPs 4 hours after nocodazole (5 µg/ml) treatment. Most of the directed motions are gone. Scale bars, 10 s (horizontal) and 10 µm (vertical), respectively. **c**. Bar graph comparing the percentage of mRNPs with directed motion during 1-minute observation before and 4 hours after nocodazole treatment. Experiment was performed on hippocampal neurons at 8 days in vitro (DIV) and more than 147 mRNPs were analyzed from 7 dendrites. Error bars represent standard error of mean (SEM).

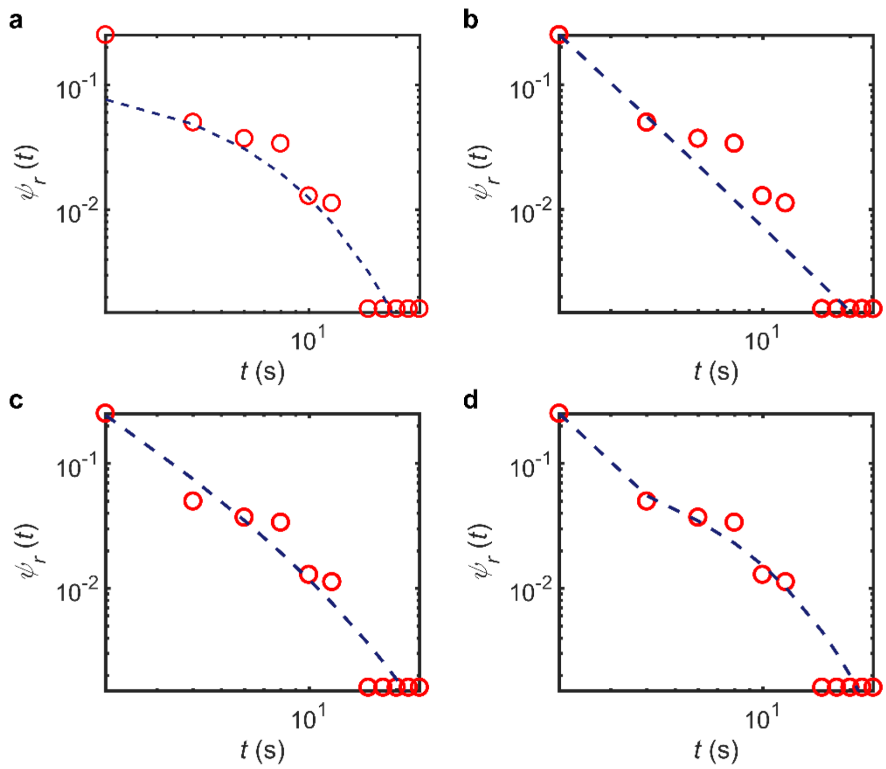

Supplementary Figure 5. Fitting curves for the run time distribution. **a.** Single-exponential fit. **b.** Power-law fit. **c.** Truncated power-law fit. **d.** Double-exponential fit. Experimental data of the run time distribution (red circles; same data shown in Fig. 2b) are displayed with the best fit curves (blue dashed line).

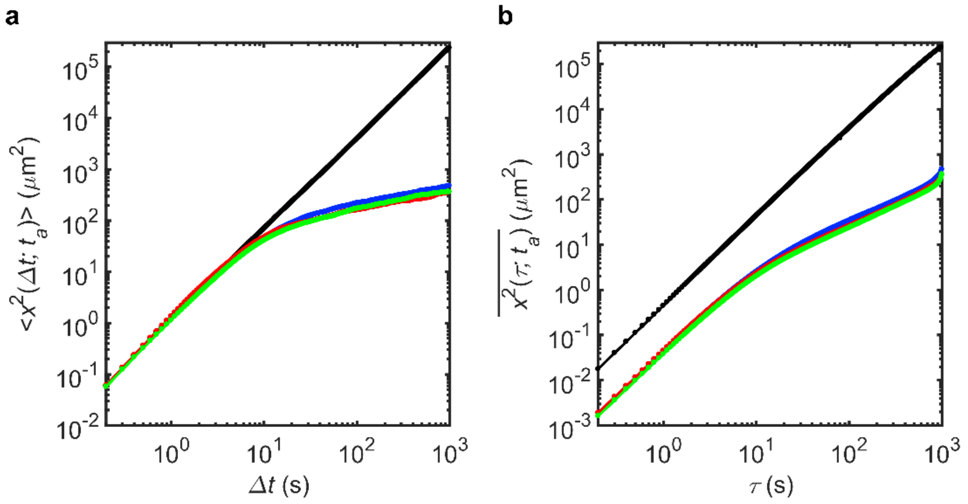

Supplementary Figure 6. MSD curves of our generalized Lévy walk model for various forms of run time pdf $\psi_r(t)$ discussed in Supplementary Note 2. **a**. EA MSD curves. **b**. TA MSD curves. In each panel, the colored solid lines represent the results for the pdf of power-law (black), power-law with an exponential cutoff (blue), single exponential (red), and double exponential (green) with the best fit parameters found in Supplementary Table 2. In the above plot, the rest time pdf was fixed to be the same power law form $\psi_s(t)$ and the aging time was $t_a = 0$.

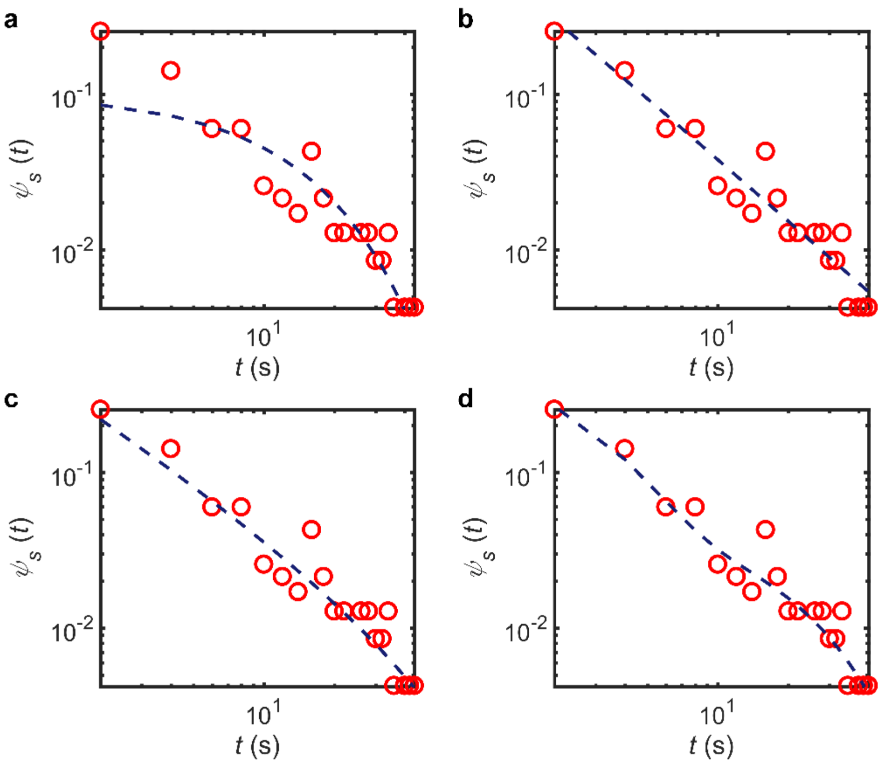

Supplementary Figure 7. Fitting curves for the rest time distribution. **a.** Single-exponential fit. **b.** Power-law fit. **c.** Truncated power-law fit. **d.** Double-exponential fit. Experimental data of the rest time distribution (red circles; same data shown in Fig. 2c) are displayed with the best fit curves (blue dashed line).

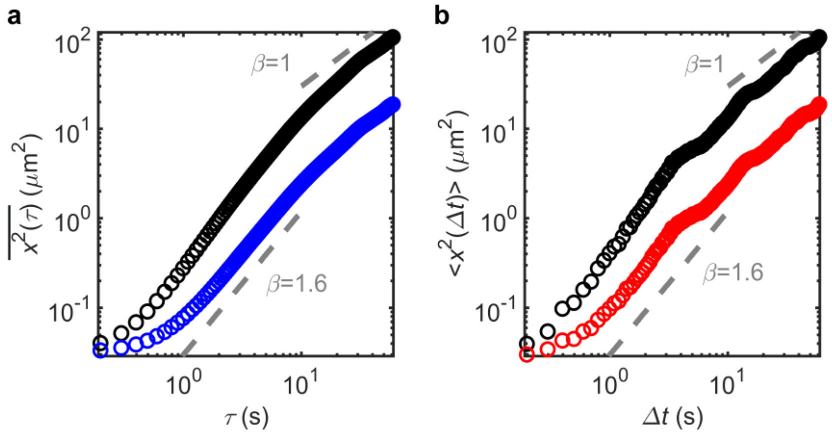

Supplementary Figure 8. Additional analysis on MSD obtained from the experiment. **a.** The averaged TA MSDs for all kymographs (blue circles; same MSD curve shown in Fig. 2e) and for a subset of kymographs showing at least one run phase (black circles). **b.** EA MSDs for all kymographs (red circles; same MSD curve shown in Fig. 2e) and for a subset of kymographs with any run phase (black circles). In both cases of total and subset analyses, consistent scaling behaviors are found. The increase of MSD amplitudes in subset analysis is simply due to the fact that only mobile mRNPs were considered. This analysis confirms that the sub-ballistic superdiffusion ($\beta \approx 1.6$) and the Fickian dynamics ($\beta \approx 1$) in Fig. 2e is the genuine dynamic feature of the mRNPs.

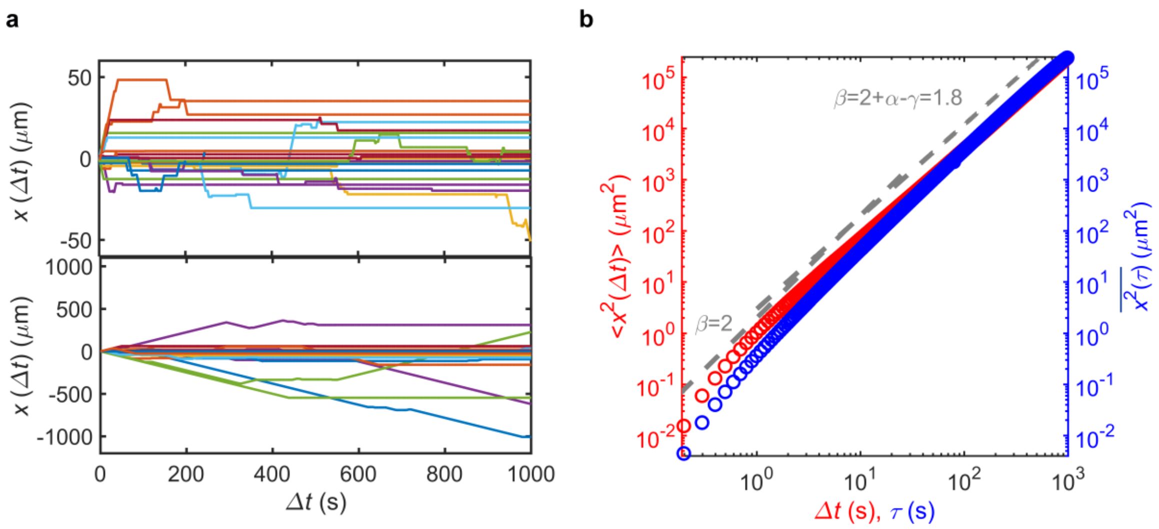

Supplementary Figure 9. The effect of truncation in the run time distribution. **a.** Simulated trajectories for the generalized Lévy walk model with truncation (upper panel) and without truncation (lower panel) in the run time pdf $\psi_r(t)$. In each panel, 23 trajectories are displayed. In the simulation, all required parameters were fixed with the values estimated from our experiment. The truncated Lévy walk shows a similar pattern of motion found in the experiment. **b.** EA MSD (red circles) and TA MSD (blue circles) of the simulated trajectories using the generalized Lévy walk model without truncation in the run time distribution.

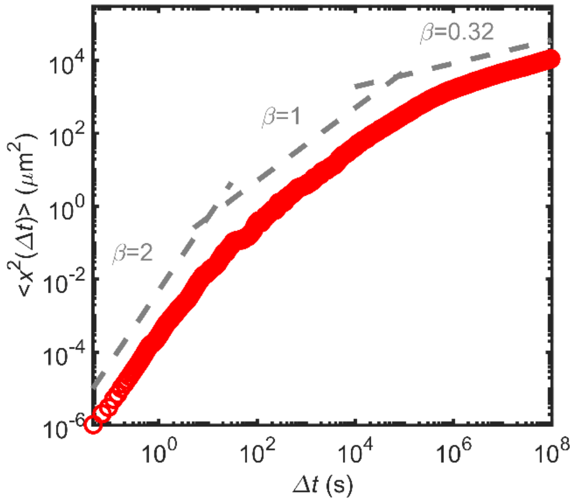

Supplementary Figure 10. The aged EA MSD for the truncated Lévy walk simulation. This plot is a long-time version of Fig. 3c. In this long-time simulation, three expected scalings are observed: (1) Ballistic regime for $\Delta t \ll t_a$. (2) Apparent Fickian regime ($\langle x^2(\Delta t)\rangle \sim t_a^{\alpha-1}\Delta t$) in the intermediate time scale $0 < \Delta t < t_a$. (3) Subdiffusive regime ($\langle x^2(\Delta t)\rangle \sim (\Delta t)^{\alpha}$) for $\Delta t \gg t_a$. The above MSD curve was obtained from 10000 simulated trajectories. The parameters used in the simulation are: α=0.32, γ=0.52, τ_r=12.5 s and t_a=10^5 s.

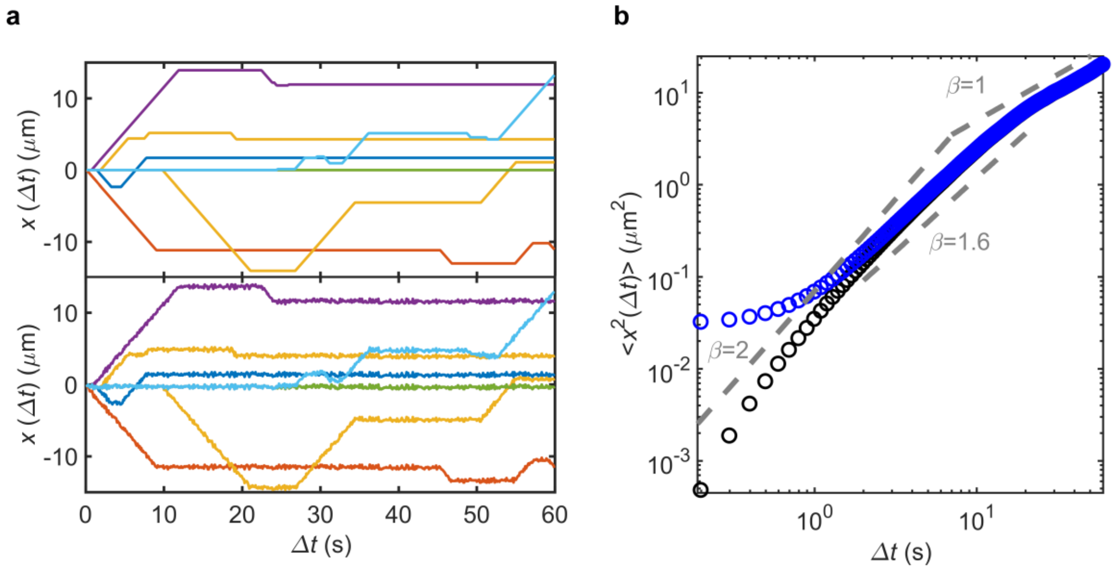

Supplementary Figure 11. The effect of noise in the trajectories. **a.** 10 sample trajectories from the simulation with aging time t_a = 100 s (upper panel) and the same trajectories added with the noise extracted from the experimental data (lower panel). **b**. EA MSD for the simulated trajectories without the noise (black circles) and EA MSD superposed with the noise extracted from the experimental data (blue circles).

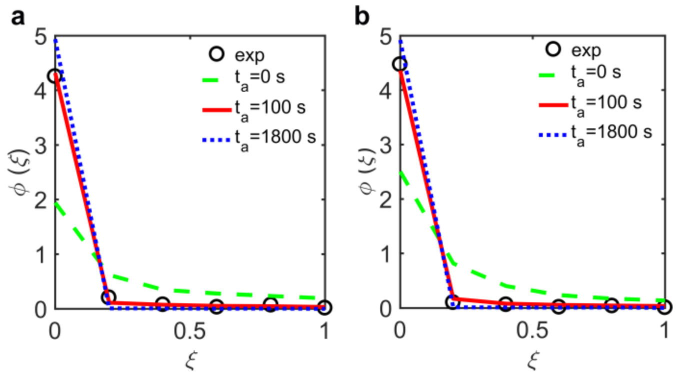

Supplementary Figure 12. Normalized amplitude scatter distribution $\phi(\xi)$ of TA MSDs as a function of rescaled variable of $\xi = \frac{\overline{x^2}}{\langle \overline{x^2} \rangle}$. **a.** $\phi(\xi)$ of TA MSDs for the lag time τ =5 s. **b.** $\phi(\xi)$ of TA MSDs for the lag time $\tau = 60$ s. In both cases, the fluctuation of TA MSDs in the experimental data is consistent with the simulation results for $t_a = 100$ s. The sharp peaks at ξ=0 indicate that there exist significant portion of trajectories having no run phase over the lag time.

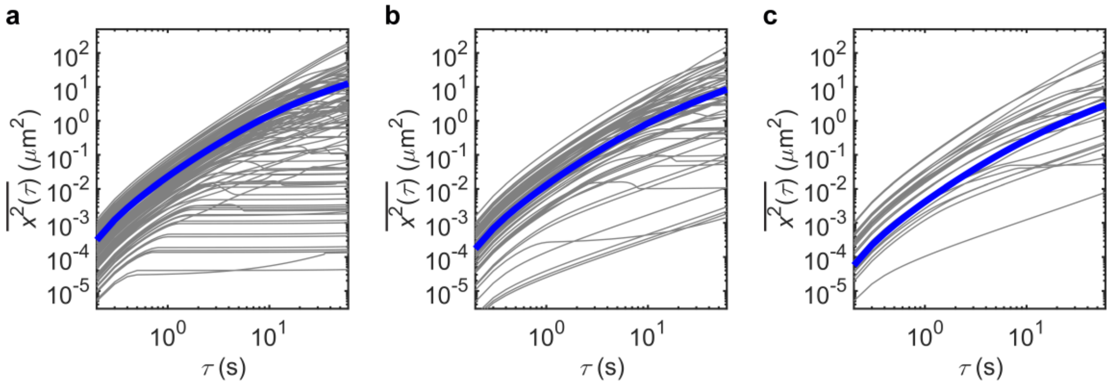

Supplementary Figure 13. TA MSD curves of the aging Lévy walk process with three different aging times. TA MSD curves with (**a**) t_a = 0 s, (**b**) t_a = 100 s, and (**c**) t_a =1800 s. Thick blue lines denote the average of 10^4 individual TA MSDs (gray). (However, in each plot only 10^2 trajectories are displayed for clarity). In **c**, the number of TA MSD curves is much less than that of **a** because the TA MSDs for the silent trajectories (without any run phase) are not visible in the log-log plot. These results clearly show that the number of silent trajectories increases with increasing t_a, which is consistent with the results in Fig. 3a.

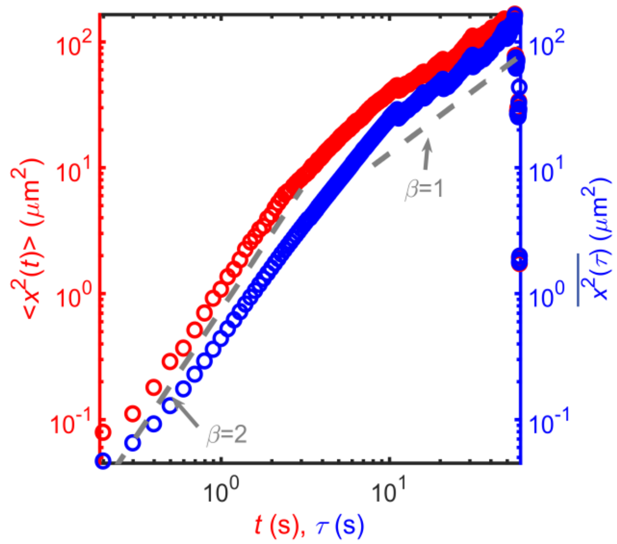

Supplementary Figure 14. Additional analysis on the aging status in the mRNP transport. Ensemble- and time-averaged MSDs for the mRNP particles were reconstructed with a subset of kymograph trajectories corresponding the cases that have at least one run event during the overall observation time. In each kymograph, the time axis was reset so that the observation ($t = 0$) starts from the first run event. This operation is expected to reverse the aging effect and, accordingly, to lead to the weak ergodicity breaking (WEB) as predicted in our Lévy walk model with $t_a = 0$ (Fig. 3b). The above plot indeed confirms this idea. Distinct from the MSD plot in Fig. 2e, in this reconstructed plot EA MSD is not the same as TA MSD. At times larger than the truncation time ($\tau_r = 12.5$ s), TA MSD shows the expected Fickian scaling due to WEB. Large scatter at the end of the curve is due to the insufficient statistics with the subset data.

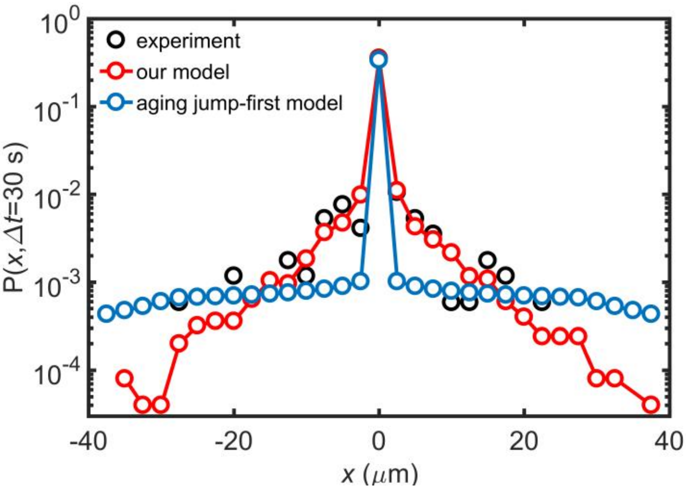

Supplementary Figure 15. Aged probability density function of mRNP particles with the theoretical curves by two distinct aging Lévy walk models. For further understanding of the aging properties of our generalized Lévy walk model, we compared the aging effect of our model with that of the jump-first model recently introduced by Magdziarz and Zorawik[1]. The latter model essentially describes the Lévy walk by a sequence of jump and rest pairs governed by the same power-law pdf. In Ref. 1, the analytic form of the aged probability density function was explicitly derived. With the same parameter conditions determined in the experiment, in this figure, the aged $P(x,\Delta t)$ of the jump-first model is plotted together with those of our model and the experiment. It is found that while the ratio of rest particles (i.e., the peak value) is explained well by both Lévy walk models, the jump-first model does not properly explain the experimental behavior for displacements larger than a few μm. This discrepancy is due to the fact that the jump-first model describes the rest and run events with the same power-law pdf whereas the experimental run and rest statistics need their own respective pdfs. The jump-first model has a significant probability having large jumps compared to our generalized Lévy walk having a truncated power-law of run times. The above result demonstrates that the aging and the profile of $P(x,\Delta t)$ sensitively depend on the statistics of run and rest events. Our Lévy walk model, which allows to deal with the run and rest events separately, is quantitatively an adequate model for the description of mRNP transport.

$P(\rightarrow \mid \rightarrow) = 0.51$	$P(\leftarrow \mid \rightarrow) = 0.49$
$P(\rightarrow \mid \leftarrow) = 0.47$	$P(\leftarrow \mid \leftarrow) = 0.53$

$\rightarrow$: Anterograde
$\leftarrow$: Retrograde

Supplementary Table 1. Conditional probabilities of the four possible directional combinations for all consecutive runs separated by a rest. Almost same conditional probabilities from 312 runs indicate that the direction of each run is independent from previous direction of run.

Model	Single-exponential		Power-law		Truncated power-law			Double-exponential			
Parameters for the best fit	*A*	τ_1	*A*	γ	*A*	γ	τ_1	*A*	τ_1	*B*	τ_2
	0.12±0.03	4.4 ±0.4	1.34 ±0.1	1.26 ±0.19	0.88 ±0.07	0.52 ±0.58	12.5 ±9.4	0.12 ±0.02	4.9 ±0.7	9.4 ±0.8	0.5 ±1.4
R^2	0.9054		0.9358		0.9466			0.9380			
RMSE	0.2548		0.2099		0.2031			0.2339			

Supplementary Table 2. Fitting parameters for the run time distribution with various models. R^2 is the coefficient of determination, which indicates the proportion of the total sum of squares explained by the model. An R^2 of 1 indicates that the regression line perfectly fits the data. Root mean square error (RMSE) is a measure of the differences between the data and values predicted by a model. An RMSE value closer to 0 indicates a better fit. The least squares error methods indicate that truncated power-law is the best model to explain the run time distribution with the highest R^2 and the lowest RMSE.

Model	Single-exponential		Power-law		Truncated power-law			Double-exponential			
Parameters for the best fit	*A*	τ_1	*A*	γ	*A*	γ	τ_1	*A*	τ_1	*B*	τ_2
	0.10 ±0.02	12.5 ±1.2	0.8 ±0.2	0.32 ±0.08	0.5 ±0.2	0.07 ±0.23	59 ±49	0.6 ±0.3	2.0 ±0.6	0.05 ±0.01	17.1 ±2.3
R^2	0.8438		0.9206		0.9258			0.9366			
RMSE	0.2022		0.1442		0.1434			0.1367			

Supplementary Table 3. Fitting parameters for the rest time distribution with various models. The least squares error methods indicate that double-exponential distribution is the best model to explain the rest time distribution with the highest R^2 and the lowest RMSE.

Supplementary Note 1: Fitting the run time distribution

The run time distribution shown in Fig. 2b was fit with the following distribution functions. The three variations of the exponentially decaying function (single-exponential, truncated power-law, and double-exponential) were chosen based on a simple physical argument that the run has a finite characteristic length in that there should be a finite number of ATPs involved in a single run event. This idea is also corroborated by a theoretical study by Müller et al.[2], which showed that the transport dynamics of a cargo via a tug-of-war competition by multiple motors leads to an exponentially truncated distribution of run length/time at large length/long time. On the other hand, run and rest occurring times can be viewed as bursting times in a complex system, where commonly observed bursting time probability density function (pdf) is of a power-law[3]. Accordingly, below we consider the four distinct distribution functions.

Single-exponential:

$$\psi_r(t) = A\exp\left(-\frac{t}{\tau_1}\right) \tag{1}$$

Power-law:

$$\psi_r(t) = \frac{A}{(t+t_0)^{1+\gamma}} \tag{2}$$

Truncated power-law:

$$\psi_r(t) = \frac{A\exp\left(-\frac{t}{\tau_1}\right)}{(t+t_0)^{1+\gamma}} \tag{3}$$

Double-exponential:

$$\psi_r(t) = A\exp\left(-\frac{t}{\tau_1}\right) + B\exp\left(-\frac{t}{\tau_2}\right) \tag{4}$$

Supplementary Fig. 5 shows the experimental data with each fitting curve. The parameters for the best fit to each distribution are shown in Supplementary Table 2. Using the least square error methods, we determined that the truncated power-law is the best model to explain the pdf of the run time.

In addition, maximum-likelihood fitting methods with goodness-of-fit tests[4] were used to determine whether truncated power-law is truly better than power-law to fit the run time distribution. Because a power-law distribution is a subset of truncated power-law distribution with $\tau_1 \rightarrow \infty$, the truncated power-law (the larger family of distribution), will always provide a better fit than the power-law (the nested or smaller family distribution). Therefore, we used a modified likelihood ratio test for the nested case [4] to determine whether the smaller family (power-law) can be ruled out. The logarithm of the likelihood ratio, R, between the power-law and truncated power-law is -14.68, indicating that truncated power-law is a better fit. The observed value of R is sufficiently far from zero and its negative sign is statistically

significant ($p < 0.01$).

Supplementary Note 2: Effects of the profile of distribution functions on the transport dynamics

As pointed out in the main text and Supplementary Fig. 9, the transport dynamics of a generalized Lévy walk process (modeling the mRNP motion) is evidently distinct depending on whether the exponential truncation exists or not in the power-law pdf of the run time. However, it turns out that for the observation time window in our experiment, the difference among the above three variations of the exponentially decaying functions is negligible in the transport dynamics. This is shown in Supplementary Fig. 6 in which the ensemble- and time-averaged MSDs of our generalized Lévy walk model for the above four fit functions are plotted with their best fit parameters. The results demonstrate that the three fit functions in the class of exponential function display very similar patterns of the ensemble- and time-averaged MSDs. This further augments our conclusion above (and in the main text) such that the exponential truncation is critical in characterizing the mRNP dynamics in our observation time scale while the details of the exponential pdf is irrelevant if it fits sufficiently well the data within the observation time window. The observed robust scaling behavior is physically understandable; at short times the dynamics is dominated by the run phase leading to the ballistic scaling while the exponent of long-time dynamics (> the truncation characteristic time) is determined by the waiting time pdf of rest events. The curvature around the cross-over time and the amplitude of the MSD curves may, in general, depend on the details of the (exponential) distribution function. Even in this case, the exponential pdfs tuned with a proper normalization and fit parameters within given time window can produce similar cross-over characteristics. Finally, we note in passing that the amplitude of the MSD curve can vary depending on whether the part of pdf whose time is shorter than our fitting range (> 2 s) is considered or not for normalization (data not shown). In this study, we used the exponentially truncated power-law distribution, which is conceptually easy to handle in the framework of Lévy walk theory.

Supplementary Note 3: Fitting the rest time distribution

The rest time distribution shown in Fig. 2c was also fit with the four different models: single-exponential, power-law, truncated power-law, and double-exponential. The results are shown in Supplementary Fig. 7 and Supplementary Table 3. All models except for a single-exponential function show reasonable fit to the data with similar coefficients of determination (R^2) and root mean square error (RMSE) values. Among the three models, we decided to use a power-law function to model the rest time distribution since it has the least number of fitting parameters. Moreover, fitting with a truncated power-law model estimates a decay constant τ_1 = 59 ± 49 s, which is close to our overall observation time of 60 s. Recently, Yoon *et al.* reported that the half-life of stationary events of β-actin mRNA is ~ 7 min using a single exponential fit to their long-term imaging data[5]. Although the rest time distribution may eventually show an exponential decay over a long period of time, the reported decay constant (~10 min) is one order longer than our total observation time (60 s). Therefore, we reasoned that a power-law function is a reasonable model to describe the rest time distribution within our observation time window.

Supplementary References

1. Magdziarz, M. & Zorawik, T. Aging ballistic Lévy walks. *Phys. Rev. E* **95**, 022126 (2017).

2. Muller, M. J., Klumpp, S. & Lipowsky, R. Tug-of-war as a cooperative mechanism for bidirectional cargo transport by molecular motors. *Proc. Natl. Acad. Sci. USA* **105**, 4609-4614 (2008).

3. Barabasi, A. L. The origin of bursts and heavy tails in human dynamics. *Nature* **435**, 207-211 (2005).

4. Clauset, A., Shalizi, C. R. & Newman, M. E. J. Power-Law Distributions in Empirical Data. *SIAM Rev.* **51**, 661-703 (2009).

5. Yoon, Y. J., Wua, B., Buxbaum, A. R., Dasa, S., Tsai, A., English, B. P., Grimm, J. B., Lavis, L. D. & Singer, R. H. Glutamate-induced RNA localization and translation in neurons. *Proc. Natl. Acad. Sci. USA* **113**, E6877-E6886 (2016).
