# Supplementary material for: Neuronal messenger ribonucleoprotein transport follows an aging Lévy walk
Source: Nat Commun. 2018 Jan 24;9:344. doi: 10.1038/s41467-017-02700-z (PMC5783941; doi:10.1038/s41467-017-02700-z)
Supplement: Supplementary file 3 — Description of Additional Supplementary File [file 41467_2017_2700_MOESM3_ESM.pdf]

Description of Additional Supplementary Files

File Name: Supplementary Movie 1
Description: **A time-lapse movie of β-actin mRNPs in a hippocampal neuron.**
Movement of β-actin mRNPs in a hippocampal neuron at 15 days in vitro (DIV) is shown. Time-lapse images were acquired at 10 fps for 60 s. While most mRNPs are stationary (i.e., not moving) for the total observation time of 60 s, some mRNPs exhibit stochastic movement in either anterograde or retrograde direction.
